# Ascorbic acid reduces Ropivacaine-induced myotoxicity in cultured human osteoporotic skeletal muscle cells

**DOI:** 10.1186/s12891-023-06702-5

**Published:** 2023-07-15

**Authors:** Maria Giovanna Scioli, Filadelfo Coniglione, Chiara Greggi, Luca Evangelista, Elena Fiorelli, Luca Savino, Amedeo Ferlosio, Eleonora Piccirilli, Elena Gasbarra, Riccardo Iundusi, Umberto Tarantino, Augusto Orlandi

**Affiliations:** 1 Institute of Anatomic Pathology, Dept. of Biomedicine and Prevention, University of Rome Tor Vergata, Via Montpellier 1, Rome, 00133 Italy; 2 Department of Clinical Sciences and Translational Medicine, University of Rome ’Tor Vergata, Rome, Italy; 3 Department of Surgical Sciences, Catholic University Our Lady of Good Counsel, Tirana, Albania; 4 Department of Orthopedics and Traumatology, PTV Foundation, Rome, Italy; 5 Department of Biomedical Sciences, Catholic University Our Lady of Good Counsel, Tirana, Albania

**Keywords:** Local anesthesia, Skeletal muscle, Myotoxicity, Oxidative stress, Myogenesis

## Abstract

**Background:**

Osteoporosis is a worldwide health issue. Loss of bone mass is a potential risk factor for fragility fractures, and osteoporotic fractures place a considerable burden on society. Bone and muscle represent a functional unit in which the two tissues are intimately interconnected. Ropivacaine is a potent local anesthetic used in clinical practice for intraoperative anesthesia and postoperative pain management, in particular for hip surgery. When injected, Ropivacaine can diffuse locally through, in particular in surrounding skeletal muscle tissue, causing dose-dependent cytotoxicity, oxidative stress and myogenesis impairment. Based on those evidences, we focused our attention on Ropivacaine-induced cytotoxicity on cultured human myoblasts.

**Methods:**

Primary human myoblasts and myotubes from healthy subjects, osteoarthritic and osteoporotic patients (OP) were cultured in the presence of Ropivacaine. In some experiments, ascorbic acid (AsA) was added as a potent antioxidant agent. Cell viability and ROS levels were evaluated to investigate the myotoxic activity and Real-Time PCR and Western blot analysis carried out to investigate the expression of proliferation and myogenic markers.

**Results:**

A dose-dependent decrease of cell viability was observed after Ropivacaine exposure in both OP myoblasts and myotubes cultures, whereas those effects were not observed in the presence of Propofol, a general anesthetic. The adding of AsA reduced Ropivacaine negative effects in OP myoblast cultures. In addition, Ropivacaine exposure also increased ROS levels and upregulated Nox4 expression, an enzyme primarily implicated in skeletal muscle ROS generation. AsA treatment counteracted the oxidant activity of Ropivacaine and partially restored the basal condition in cultures. Positive myogenic markers, such as MyoD and Myf5, were downregulated by Ropivacaine exposure, whereas myostatin, a negative regulator of muscle growth and differentiation, was upregulated. The phenotypic deregulation of myogenic controllers in the presence of Ropivacaine was counteracted by AsA treatment.

**Conclusions:**

Our findings highlight the oxidative stress-mediated myotoxic effect of Ropivacaine on human skeletal muscle tissue cell cultures, and suggest treatment with AsA as valid strategy to mitigate its negative effects and allowing an ameliorated functional skeletal muscle recovery in patients undergoing hip replacement surgery for osteoporotic bone fracture.

**Supplementary Information:**

The online version contains supplementary material available at 10.1186/s12891-023-06702-5.

## Introduction

Osteoporosis is considered by the World Health Organization to be one of the most critical health problems after cardiovascular disease [1]. Osteoporosis is a condition characterized by an impairment of both the structural properties and quality of bone, and predisposes patients to an increased risk of fragility fracture [2]. Based on a rapidly expanding body of evidence, osteoporosis is likely caused by complex interactions between local and systemic regulatory factors of bone cell function [3]. The skeletal muscle tissue primarily interacts with bone, representing together a functional unit in which the two tissues are anatomically intimately connected, both also from a biomechanical and biochemical point of view. Therefore, bone changes are reflected in skeletal muscle changes and vice versa [4]. The biochemical connection between the two tissues is mediated by myokines and osteokines, molecules released by muscle and bone respectively, under physiological and pathophysiological conditions, which can exert a regulatory effect on a wide variety of organs and tissues, including bone and muscle themselves [5]. The connection between the two tissues is also biomechanical in that bone adapts its morphology and strength to the long-term load exerted by muscle during physical activity, since osteocytes represent the mechanoreceptors of bone tissue [6]. The most significant physiopathological changes that occur in bone and muscle are those brought about by aging [7]. Among those, oxidative stress is preponderant and, according to the literature, high levels of reactive oxygen species (ROS) cause detrimental effects on the health of the musculoskeletal system [8]. Indeed, ROS generation negatively affects vital cellular processes such as autophagy, differentiation and induces proliferation arrest and cell death through the activation of several molecular pathways [9]. In the musculoskeletal context, oxidative stress from ROS accumulation exerts negative effects on myogenic differentiation and survival, leading to loss of muscle mass and function [10]. Among the enzymes responsible for the generation of ROS, NADPH oxidase 4 (Nox4) appears to be highly expressed in skeletal muscle tissue, and plays a primary role in myogenic differentiation and oxidative stress, when overexpressed [8, 11]. Ropivacaine is a potent local anesthetic, frequently used in the clinical practice for intraoperative regional anesthesia and postoperative pain management, given its potent long-lasting analgesic effect [11]. After its injection in treatment area, Ropivacaine diffuses through numerous surrounding tissues including skeletal muscle, causing a dose-dependent cytotoxic effect [12]. It has been suggested that Ropivacaine-induced myotoxicity is due to an increase of intracellular Calcium levels, which is responsible for the acute cytotoxic effect. Such increase stimulates energy-intensive processes, which result in an altered cellular homeostasis [13]. As reported by Galbes and colleagues, local anesthetics also appear to cause increased levels of ROS, which are responsible for caspase-induced cell apoptosis, partially reversed by the treatment with N-acetylcysteine, which has anti-oxidant properties [14]. As mentioned earlier, osteoporosis and resulting fragility-induced bone fractures are a relevant public health problem, placing significant socioeconomic burdens on those affected and the community. According to literature reports, local anesthetics are capable of exerting a cytotoxic effect on bone, muscle, and joints, in a concentration- and time-dependent manner. These drugs have been shown to decrease cell viability, leading to cell death by autophagy, necrosis, and apoptosis [15]. Specifically, it has been observed that administration of local anesthetics results in histological changes including: degeneration and dissolution of sarcoplasmic reticulum, presence of edema, inflammatory infiltrate in surrounding areas, and necrosis [16]. The result of these alterations in muscle tissue could result in the release of molecules that, given the intimate anatomical connection between muscle and bone, could cause damaging effects on the latter, thereby compromising the normal bone healing process and the patient’s subsequent rehabilitation course. The aim of the present study was to investigate the effect of Ropivacaine on proliferation, differentiation, and oxidative stress levels in cultured human myoblasts isolated from subjects undergoing surgical intervention for high-energy fracture, patients undergoing arthroplasty surgery for coxarthrosis and patients undergoing surgery for hip fragility fracture. We also evaluated the effect in cell cultures of co-treatment with Ascorbic Acid (AsA), also known as Vitamin C, an important antioxidant able to prevent oxidation of numerous substances [17].

## Materials and methods

### Patients

We enrolled 28 patients who underwent hip surgery in the Orthopedic Department of “Tor Vergata” University Hospital, between 2016 and 2018. Specifically, we enrolled 10 osteoporotic older patients (OP) who underwent hip arthroplasty for subcapital fractures of the femur (age 78.58 ± 6.3 years; mean ± standard error of the mean (SEM)) and 10 osteoarthritic older patients (OA) who underwent hip arthroplasty for coxarthrosis (age 73.44 ± 8 years); 8 patients undergoing femoral osteosynthesis surgery for high-energy fracture (healthy) were also enrolled as control subjects (age 44.34 ± 2 years). Exclusion criteria were history of cancer, myopathies or other neuromuscular diseases or chronic administration of corticosteroid for autoimmune diseases (more than 1 month), diabetes, alcohol abuse, and HBV, HCV, or HIV infections. The main characteristics of OP, OA and healthy patients are described in Table 1.

**Table 1:** Clinical characteristics of OP, OA patients and Healthy subjects

Characteristics	OP (n = 10)	OA (n = 10)	Healthy (n = 8)
Age (years)	78.58 ± 6.3	73.44 ± 8	44.34 ± 2
BMI (Kg/cm^2^)	23.4 ± 4.7	24.2 ± 2.3	24.4 ± 3.1
t-score L1–L4	−1.5 ± 1.3	0.78 ± 1.2	0.8 ± 1.0
t-score total femur	−1.8 ± 1.3	0.8 ± 1.3	1.0 ± 0.7
t-score femoral neck	−2.1 ± 1.2	0.7 ± 1.4	1.2 ± 1.0
BMD L1-L4 (g/cm^2^)	1.0 ± 0.2	1.1 ± 0.2	1.2 ± 0.1
BMD total femur (g/cm^2^)	0.8 ± 0.3	0.9 ± 0.2	1.1 ± 0.2
BMD femoral neck (g/cm^2^)	0.6 ± 0.1	0.9 ± 0.3	1.0 ± 0.1
Calcium (mg/dL)	8.2 ± 0.5	8.3 ± 0.4	8.5 ± 0.5
PTH (pg/mL)	99.6 ± 65	75.2 ± 19	73.3 ± 17.2
25-(OH)-Vit D (ng/mL)	16.3 ± 10	24.1 ± 4.2	25.2 ± 3.1

BMI, body mass index; BMD, bone mineral density, PTH, parathyroid hormone; 25-(OH)-Vit D, 25-hydroxyvitamin D

### Bone mineral density evaluation

Bone mineral density (BMD) was evaluated with a Lunar DXA apparatus (GE Healthcare, Madison, WI, USA). Lumbar spine (L1–L4) and femoral (neck and total) scans were performed, and BMD was measured according to manufacturer’s recommendations [18]. Dual-energy X-ray absorptiometry measures BMD (in grams per cm^2^), with a coefficient of variation of 0.7%. For patients with fragility fractures, BMD was measured on the uninjured limb. For OA patients, measurements were performed on the non-dominant side, with participants supine on an examination table with their limbs slightly abducted. DXA exam was performed one day before surgery for OA patients, and 1 month after surgery for OP patients. The results were expressed as T-scores.

### Cell culture, treatments and viability assay

Human myoblast cells were isolated by explant from vastus lateralis muscle biopsies of patients underwent hip surgery and grown on gelatin matrix (G9391, Merck Life Science S.r.l., Milan, Italy) in complete growth medium: Ham’s F14 (L0138-500, Biowest, Nuaillé, France) supplied with 15% FBS (F7524, Merck) insulin 1 mg/ml (I9278, Merck), 2mM L-glutamine (G7513, Merck), 100U/mL penicillin-100 µg/ml streptomycin (P4333, Merck) and 0.25 µg/mL Amphotericin B (A2942, Merck), FGF 5 µg/ml (RP-8628, Thermo Fisher Scientific, MA, USA) and EGF 10 µg/ml (01-AA060-0010, ISOkine, ORF Genetics, Island) [1, 19]. Myoblasts were characterized for MyoD expression at passage 1 (after the explant passage 0). Then, a second characterization was performed in parallel to the seeding for the experiments (strictly at passage 3–4). For myotubes, differentiation was induced at 90% of confluence of myoblast cultures, strictly at passage 3–4, by adding culture medium without growth factors with only human insulin, for 15 days. Differentiation was continuously monitored by light microscope and confirmed by MYH1 upregulation.

Briefly, cells were plated in 96-well plates at a density of 8000 cell/cm^2^ with 100 µl of complete culture. Then, after anesthetic treatments at different concentrations (0-18mM Ropivacaine for 2 h and 0-1000µM Propofol) for 48 h) and next 24 h of recovery, cell viability was analyzed by using CCK8 assay (96,992, Merk) for IC50 determination. Ascorbic acid (100 µM) was pre-incubated over-night (prior the Ropivacaine adding at 11,5 mM) and/or post-incubated during the recovery time (after Ropivacaine treatment). Results were expressed as the mean of three independent experiments performed in triplicate.

### Reactive oxygen species detection

ROS level in myoblasts were measured by 5-(and-6)-chloromethyl-2’,7’-dichlorodihydrofluorescein diacetate, acetyl ester (CM-H2DCFDA) fluorescence method (C6827, Thermo Fisher Scientific), as previously reported [20]. Briefly, cells were seeded in 96-well chambers at a density of 8000 cell/cm^2^ with 100 µl of complete culture medium. Then, at the same time with Ropivacaine (11,5mM) and AsA co-treatment, cells were incubated with 10 µM CM-H2DCFDA at 37 °C in the dark for 30 min. Dichlorofluorescein fluorescence was measured by a fluorescence microtiter plate reader (Glomax Explorer, Promega, Milan, Italy). Results were expressed as the mean of three independent experiments performed in triplicate.

### RNA extraction and real-time PCR

Total RNA was isolated from treated and untreated cells using Tri Reagent (Merck), according to the manufacturer’s instructions. RNA (2 µg) was reverse-transcribed using the SuperScript III (Invitrogen, Thermo Fisher Scientific, MA, USA). Real-time PCR was carried out using SYBR Green (BioRad, Hercules, California, USA) and gene expression was analyzed using the following primer pairs: Myf5 forward 5’-TGAGAGAGCAGGTGGAGAACTACT-3’ and reverse 5’-AGACAGGACTGTTACATTCGGGCA-3’; MyoD forward 5’- TGCCACAACGGACGACTTCTATGA-3’ and reverse 5’-AAGTGCGAGTGCTCTTCGGGTTT-3’, respectively. Real-time PCR was performed on Applied Biosystem StepOnePLus (Thermofisher Scientific) as reported [21] and gene expression normalized to GAPDH. Target gene expression levels were calculated using the comparative ΔΔCT method. Fold change was considered significant for values > 2.0 and < 0.5. Results were expressed as the mean of three independent experiments performed in triplicate.

### Western blot analysis

After extraction and quantification of total cell lysates by Bradford method, 30 µg of protein sample were separated on 10% sodium dodecyl sulfate polyacrylamide gel (SDS–PAGE) [22]. Then, proteins were elettroblotted onto nitrocellulose membrane [22] and incubated with a monoclonal mouse anti-GDF8/Myostatin antibody (1:1000, ab203076, Abcam, United Kingdom), a monoclonal rabbit anti-Nox4 antibody (1:200, sc-518,092, Santa Cruz Biotechnology, California, USA) and a rabbit monoclonal anti-GAPDH antibody (1:000, #2118, Cell Signaling, Massachussets, USA), followed from horseradish peroxidase conjugate goat anti-rabbit or anti-mouse IgGs (ab205718 or ab205719, Abcam, United Kingdom). Specific complexes were revealed and quantified, and densitometric blot analysis were performed in three independent experiments.

### Microscopic examination

Small skeletal muscle biopsies from patients underwent to surgery were fixed in 4% paraformaldehyde for 24 h and paraffin embedded. 3 μm-thick sections were stained using haematoxylin and eosin (H&E) (05-06002, Bio-Optica, Milan, Italy) to perform histomorphometric analysis. Ten microscopic images, randomly selected, were evaluated for each biopsy sample. Images were acquired at 20 x magnification using a Nikon upright microscope ECLIPSE CiS/ (Nikon Corporation, Tokyo, Japan) connected to a Nikon digital camera. Image analysis was performed using NIS-Elements software (5.30.01; Laboratory Imaging, Prague, Czech Republic), according to the manufacturer instructions. For histomorphometric analysis, the diameter of 200 fibers per section was measured.

### Statistical analysis

Statistical analyses were performed using STATA software version 20. Significant differences among qualitative variables were calculated by using Fisher test. Continuous variables (including systemic blood molecule, protein, and gene expression levels) were expressed as mean ± SEM (standard error of mean). Unpaired t-test (Welch corrected) was utilized to analyze the data between two groups, while one-way ANOVA or Kruskal-Wallis’s test followed by Bonferroni correction or Dunn test was applied to compare more than two groups. Differences were considered significant when a p value < 0.05 was obtained by comparison between the different groups.

## Results

### Ropivacaine influences human OP myoblast viability

**Fig. 1:**
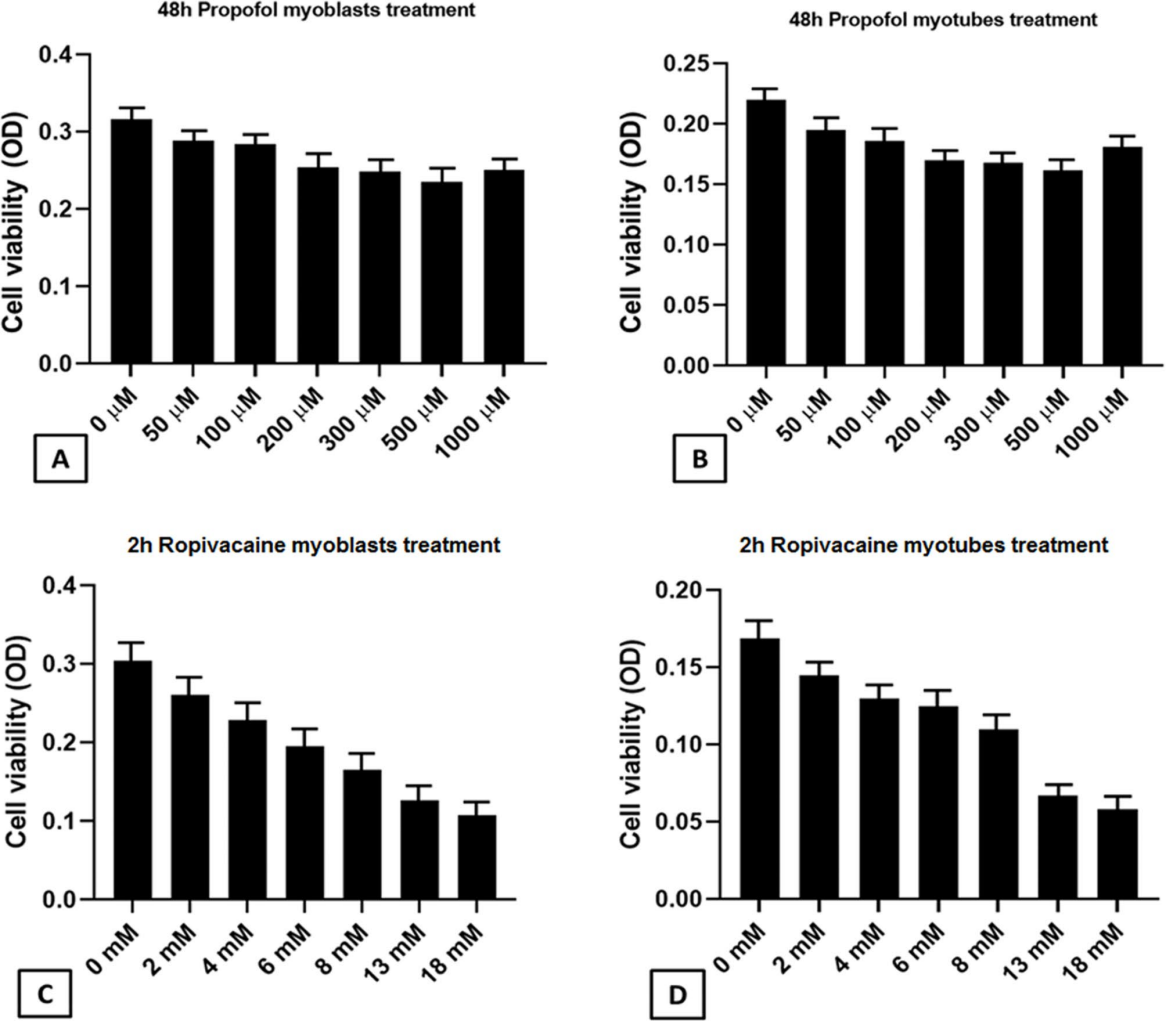
Effects of Ropivacaine and Propofol on human OP myoblast and myotube viability. **A, B)** Histograms showing CCK8 assay (viability test) on human OP myoblasts and myotubes under increasing concentration of Propofol (0–1 mM). **C, D)** Histograms showing CCK8 assay (viability test) on human OP myoblasts and myotubes under increasing concentration of Ropivacaine (0–18 mM). Results were expressed as mean ± SEM. Abbreviation: OD, optical density

Cultured human myoblasts and myotubes from OP patients were exposed to increasing concentration of Ropivacaine (local anesthestic) and Propofol (general anesthestic) for IC50 determination. After 24 h-recovery, cells showed different viabilities in the presence of the two anesthetics in culture. In particular, increasing concentrations of Propofol slightly influenced cell survival of both myoblasts and myotubes. At the highest Propofol concentration of 1mM, myoblasts and myotubes showed even a recovery of viability compared lower concentrations (Fig. 1A and B), according to the literature [23]. Instead, the adding of Ropivacaine induced an evident dose-dependent reduction of survival in both myoblast and myotube cultures (IC50 = 11.5 mM; Fig. 1C and D).

### Ropivacaine affect myoblast viability OP but not of healthy and osteoarthritic cultures

**Fig. 2:**
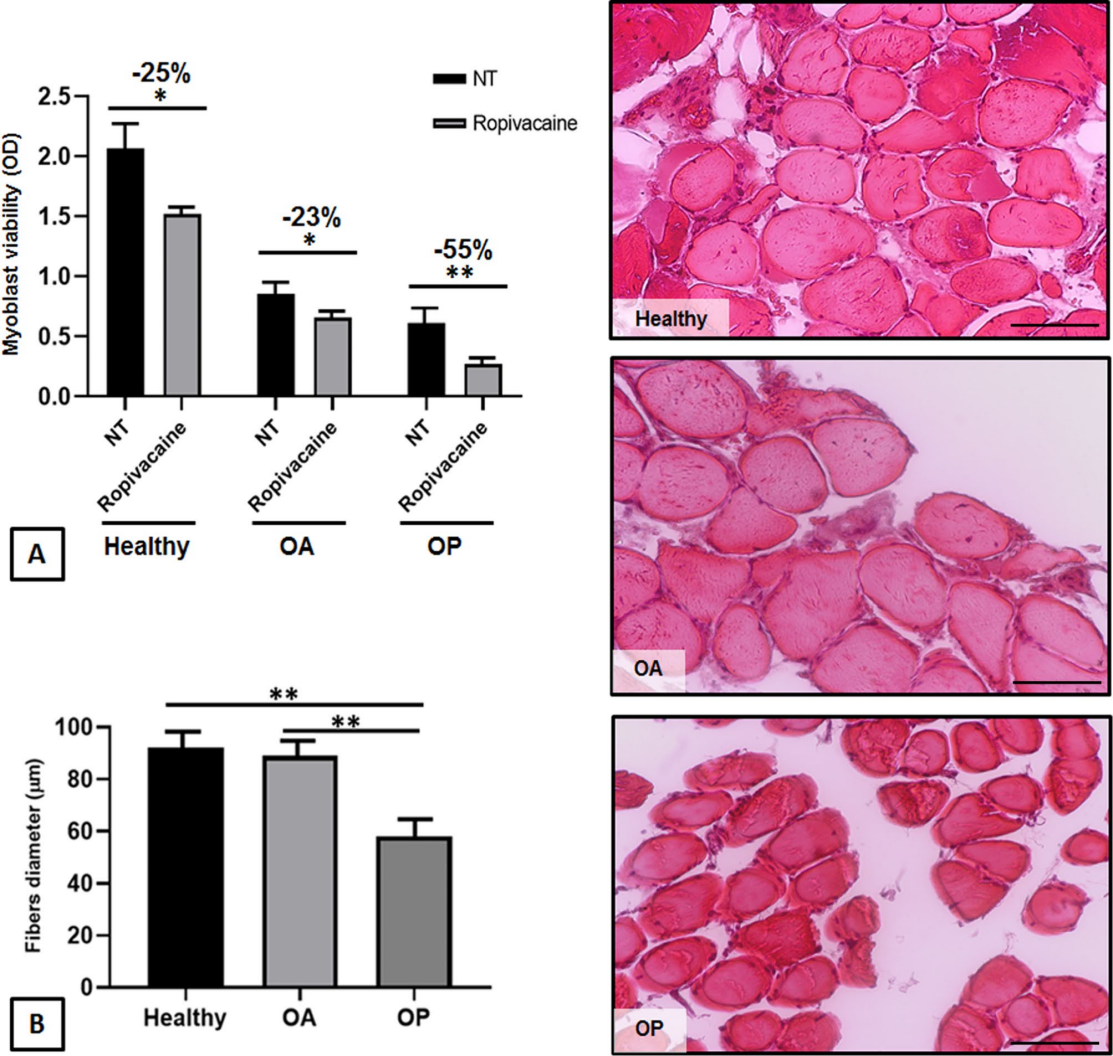
Ropivacaine effects on myoblast viability. **(A)** Histogram showing CCK8 assay (viability test) on human myoblasts from healthy, osteoarthritic (OA) and OP (OP) patients treated or not with Ropivacaine (11.5 mM). **(B)** Representative images and histomorphometric analysis of H&E-stained section of skeletal muscle from healthy, osteoarthritic and OP subjects showing fiber diameters for each group. Scale bar = 100 μm. Results were expressed as mean ± SEM. t-test: * and ** indicate p < 0.05 and p < 0.01, respectively. Abbreviations: OD, optical density; NT, not treated

Established the cytotoxicity of Ropivacaine in both OP myoblasts and myotubes, we wanted to verify if that effect was also observed in healthy and OA myoblasts cultures. OP myoblasts in the presence of Ropivacaine (11.5 mM) demonstrated the lowest cell viability after treatment (about 50% reduction; p < 0.01) compared healthy and OA myoblasts. The latter showed a better recovery, even if cell viability was significantly reduced by Ropivacaine exposure compared to control groups (about 25% reduction; p < 0.05) (Fig. 2A). This is the results of an already strictly compromise condition of OP skeletal muscle, as also demonstrated by the histomorphometric analysis (Fig. 2B). In fact, measurement of skeletal muscle fiber diameter showed significant differences between OP, OA and healthy subjects. The diameter of muscle fibers was classified into three different ranges: 40–69 μm, 70–99 μm and 100–150 μm. For the experimental group of OP patients, the percentage of fibers in the 40–69 μm range was 79%, 10% in the 70–99 μm range and 3% in 100–150 μm range. For OA patients, 20% of muscle fibers was in the 40–69 μm range, 75% in the 99 − 70 μm range and 60% of the fibers was in the 100–150 μm range. For healthy subjects, 30% of the muscle fibers were in the 40–69 μm range, 70% in the 70–99 μm range, and 80% was in the 100–150 μm range. In fact, OP skeletal muscle fiber diameter (58 μm ± 9.3) was significantly lower than that of OA patients (89 μm ± 7.2) and healthy subjects (92 μm ± 9.9; healthy vs. OP, p < 0.01; OA vs. OP, p < 0.01). In contrast, no significant difference was found between OA and healthy skeletal muscle values. Those findings suggested that skeletal muscle in OP patients is qualitatively worse than that of healthy and OA subjects; the latter showed a comparable quality.

### Ascorbic acid improves human OP myoblast recovery counteracting Ropivacaine mitotoxicity

**Fig. 3:**
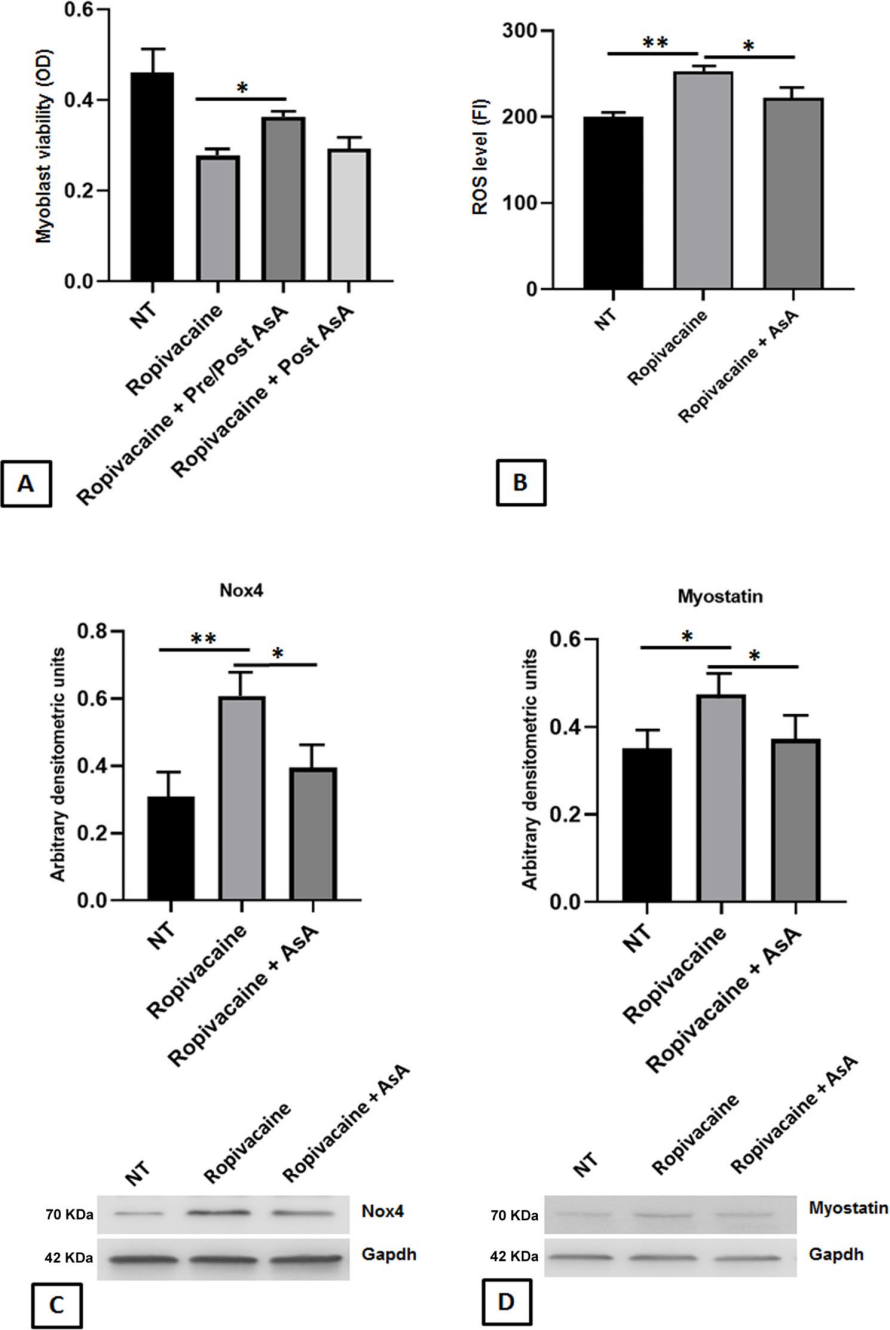
Ascorbic Acid counteracts Ropivacaine-induced effects on OP myoblast viability and oxidative stress. **(A)** Histogram showing CCK8 assay on human myoblasts from OP patients treated or not with Ropivacaine alone or with ascorbic acid (AsA, 100 µM), as pre and post treatment, before and after Ropivacaine adding (Ropivacaine + Pre/Post Asa), or only post treatment after Ropivacaine adding (Ropivacaine + Post AsA). **(B)** Bar graph showing ROS level (CM-H2DCFDA assay) in human myoblasts from OP patients treated or not with Ropivacaine alone or with ascorbic acid (100 µM) as pre and post treatment, before and after Ropivacaine adding (Ropivacaine + AsA). **C** and **D)** Representative images of blot and densitometric analysis of Nox4 and Myostatin expression of human myoblasts from OP patients treated or not with Ropivacaine alone or with ascorbic acid (AsA, 100 µM). Images do not show full-length membranes because membranes were cropped before antibody incubation and the acquisition was only performed digitally. Results were expressed as mean ± SEM. t-test: * and ** indicate p < 0.05 and p < 0.01, respectively. Abbreviations: OD, optical density; NT, not treated; FI, fluorescence intensity

Then, we investigated if the toxicity effects of Ropivacaine, reported to be mitotoxic [24, 25], could be counteracted by an antioxidant agent, such AsA. As reported in Fig. 3A, the adding of AsA at 100 µM before and after Ropivacaine exposure (Pre/Post) in cultures significantly improved OP myoblast recovery of cell viability (from about 40–20% of the reduction; p < 0.05). The adding of AsA only during the 24 h-recovery (as post-treatment after Ropivacaine adding) did not ameliorate cell recovery; then, in all the following experiments AsA was added before and after Ropivacaine treatment. The mitotoxic effect of Ropivacaine was also confirmed by CM-H2DCFDA assay, that revealed an increased production of ROS in OP myoblasts compared with control group (Fig. 3B; p < 0.01). AsA also counteracted Ropivacaine-induced ROS accumulation (p < 0.05 vs. Ropivacaine alone). In parallel, the overproduction of ROS associated with an increased expression of Nox4 (Fig. 3C; p < 0.01), one of main source of ROS in the skeletal muscle [8]. The overexpression of Nox4 was significantly prevented by the adding of AsA (Fig. 3C; p < 0.05), strongly supporting the hypothesis the oxidative stress contributes to the reduction of cell viability.

### Ascorbic acid improves human OP myoblast proliferation and miogenesis affected by Ropivacaine exposure

**Fig. 4:**
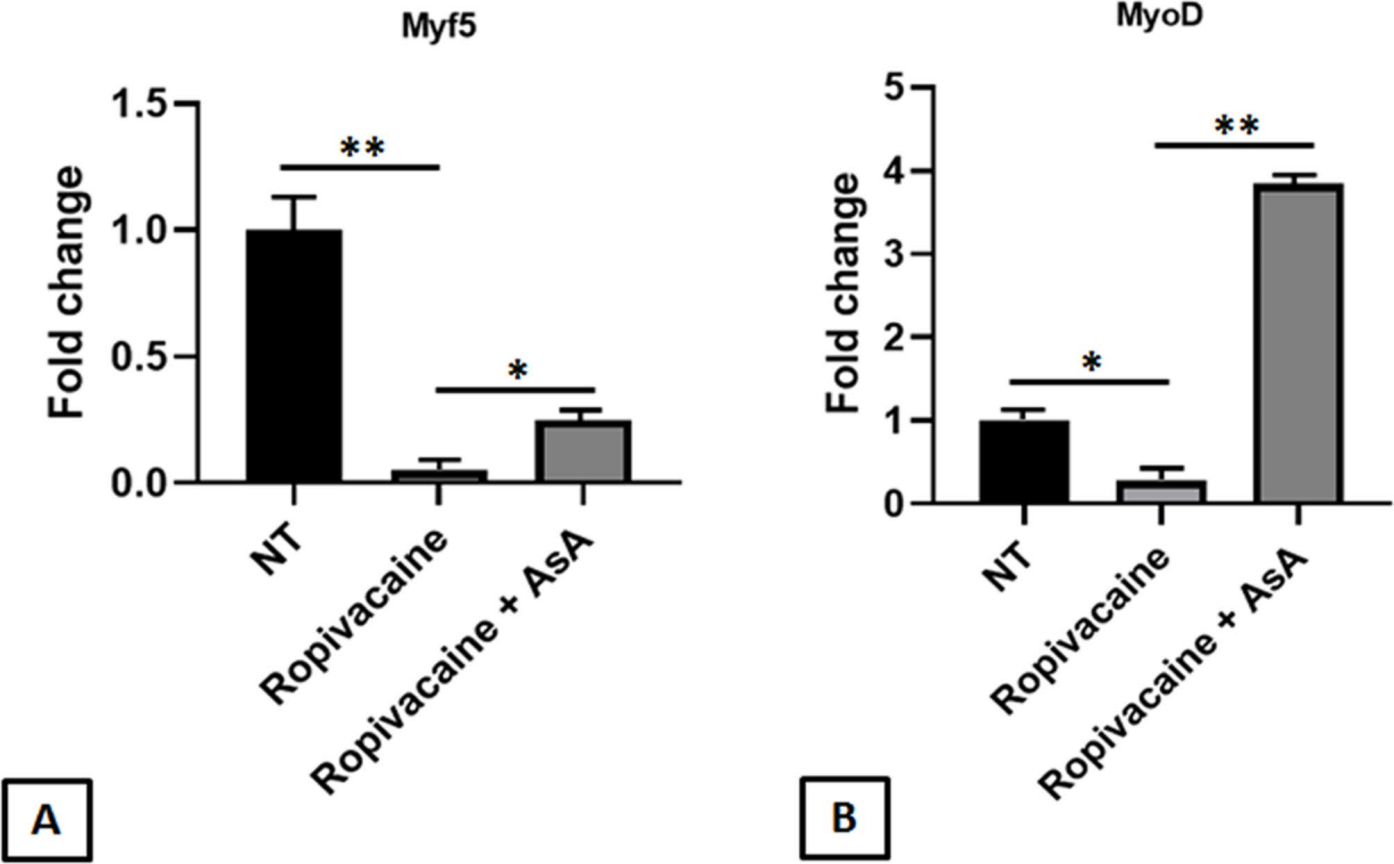
Ropivacaine affects human OP myoblast proliferation and myogenesis. Gene expression analysis on RNA extracted from human OP myoblasts treated with Ropivacaine shows decreased levels of the proliferation and myogenic markers Myf5 **(A)** and MyoD **(B)**. Ascorbic Acid (AsA) treatment counteracts the Ropivacaine-induced downregulation of these markers. Changes in gene expression were calculated using the comparative ΔΔCT method. Fold change is considered significant for values > 2.0 and < 0.5. Results were expressed as mean ± SEM. t-test: * and ** indicate p < 0.05 and p < 0.01, respectively

Next, we found that the toxicity of Ropivacaine also induced the increase of Myostatin expression, a negative regulator of proliferation and myogenesis (Fig. 3D; p < 0.05). The increase of Myostatin expression was counteracted by the adding of AsA (Fig. 3D; p < 0.05). In addition, Real-Time analysis showed a Ropivacaine-induced downregulation of myoblast determination protein 1 (MyoD) and myogenic factor 5 (Myf5) transcripts, two important positive regulators of myoblast proliferation and differentiation (Fig. 4A and B; p < 0.01 and p < 0.05, respectively). Myf5 and MyoD gene expression inhibition was partially prevented (Myf5) or even overturned (MyoD) by AsA to human OP myoblast cultures (p < 0.05 and p < 0.01, respectively).

## Discussion

Osteoporosis is a worldwide health issue and is more common in women than in men. Loss of bone mass is a potential risk factor for fragility fractures, and fragility fractures place a considerable burden on society [26]. Bone tissue greatly interact with skeletal muscle tissue [4]. The ‘bone–muscle’ unit is the site of privileged exchanges in which they interplay and communicate via paracrine and endocrine signals to coordinate development and adapt their response to loading and injury from embryologic stages to involution [27]. The most commonly used local anesthetics in orthopedics and traumatology are Lidocaine, Bupivacaine and Ropivacaine, whose damaging effects on bone, muscle and cartilage are well known [15]. Ropivacaine is a potent local anesthetic, used very frequently in clinical practice for intraoperative anesthesia and postoperative pain management, in particular for hip arthroplasty [11]. During its pathway from the injection site to the treatment area, Ropivacaine diffuses through numerous tissues including muscle tissue. Our in vitro results confirmed the previously reported cytotoxic and mitotoxic effects of Ropivacaine in fibroblast cultures [28], parallel to ROS overproduction and increased oxidative stress [8]. We focused our attention on the effects of Ropivacaine exposure on myoblasts (progenitor cells) and myotubes (differentiated cells) in order to understand the impact on cell behavior. We documented a relevant and dose-dependent impact on viability after 24 h-recovery both in myoblast and myotube cultures, especially in those from OP patients, whose condition is already strictly compromise and often patients undergo to hip surgery under local anesthesia, as also documented from skeletal muscle atrophy. Instead, the adding as control of Propofol, a general anesthetic, did not induce such impairment of cell viability. Based on the reported evidence that Ropivacaine exerts cytotoxic and mitotoxic effects, we tried to counteract its negative activity by adding a potent antioxidant agent, AsA. Since cell viability impairment associated to an increased ROS production, we investigated if that antioxidant may mitigate Ropivacaine negative effects on cell survival. Interestingly, the antioxidant efficacy of AsA was reached only when added prior and after Ropivacaine exposure, not only with a single pre or post-exposure treatment. That finding can be explained by the strong oxidant activity exerted by Ropivacaine inducing an intracellular signaling cascade that cannot be effectively counteracted only after its triggering but needs a preventive AsA treatment that likely buffers and prepare cells to an efficient response. In parallel, we also detected with Ropivacaine exposure an increase in ROS levels counteracted by the adding of AsA, supporting the evidence that Ropivacaine affects cell viability, at least in part, by its oxidant activity. In this regard, ROS increased level was also accompanied by Nox4 overexpression, one of the main source of ROS in endothelial cells (Stasi ATVB 2010) and skeletal muscle [8]. Nox4 upregulation in OP myoblast cultures was counteracted by the antioxidant activity of AsA.

**Fig. 5:**
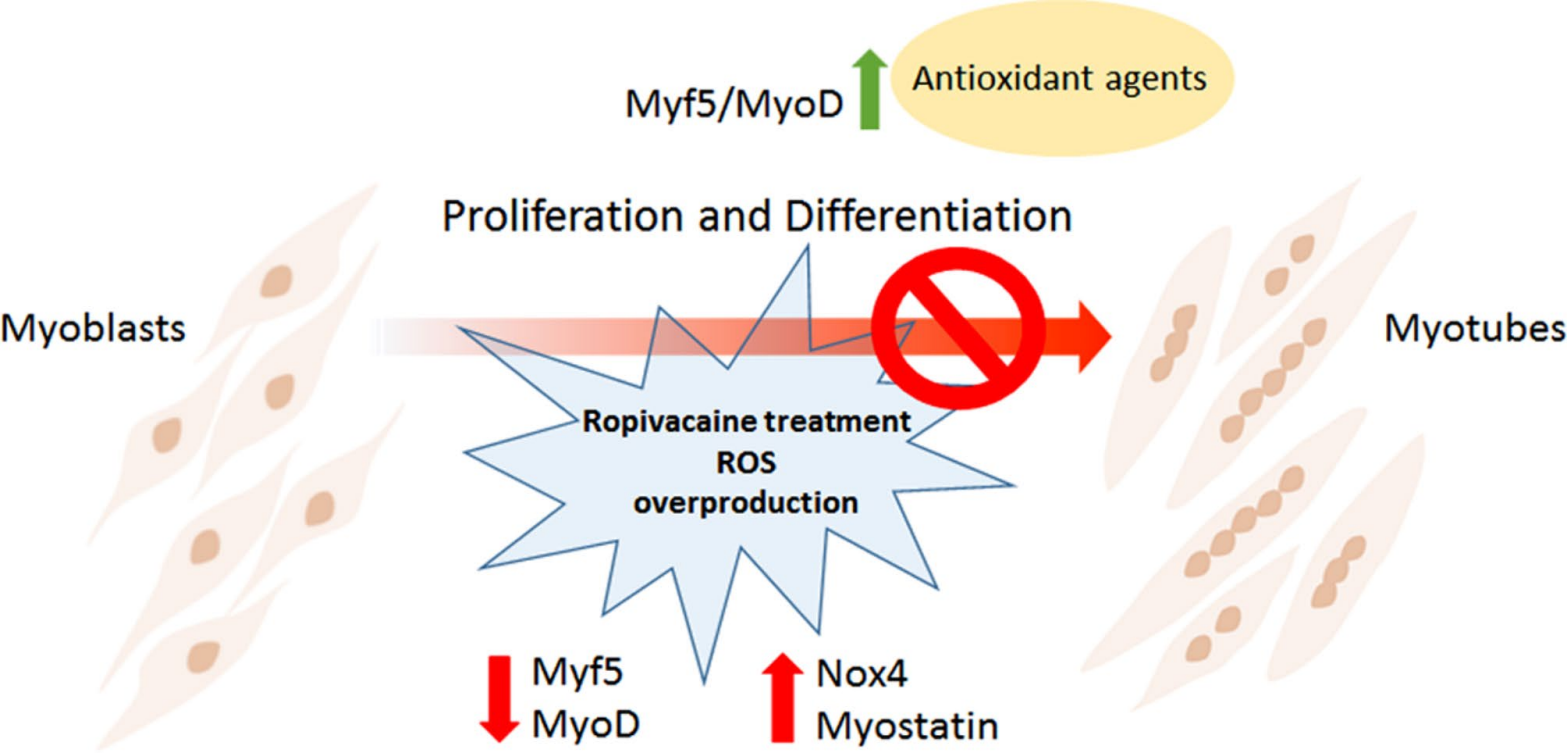
Effects of Ropivacaine and AsA treatments on human myoblasts. Graphical summary showing the inhibitory effect of Ropivacaine on myoblast proliferation and differentiation (downregulation of Myf5 and MyoD; upregulation of Nox4 and Myostatin) and the recovery effect of antioxidant agent (AsA) treatment (pre and post Ropivacaine exposure) on myoblast growth and myogenesis (upregulation of Myf5 and MyoD; downregulation of Nox4 and Myostatin)

As previously reported, myogenesis is an important process to maintain muscle homeostasis and the physiological function of skeletal muscle. Accumulating evidence suggests that oxidative stress plays a key role in myogenesis and skeletal muscle physiology and pathology [8]. So, we investigated the expression of important negative and positive regulators of myoblast proliferation and differentiation after Ropivacaine treatment. We detected a Ropivacaine exposure-induced myostatin upregulation, a negative regulator of cell proliferation and myogenesis. At the same time, myoblast transcription of MyoD and Myf5, positive differentiation controllers, were downregulated by Ropivacaine exposure. The adding of AsA reversed the Ropivacaine-induced myogenic gene deregulation and restored MyoD and Myf5 expression (Fig. 5). AsA positive effects on human myogenic differentiation has been already reported in Piaractus mesopotamicus (fish) myoblasts [29] and mouse C2C12 myoblast cells [30], in which AsA increased the activity of the antioxidant enzyme superoxide dismutase and the expression of myogenin, an important myogenic marker. According to literature studies, compared with other more commonly used local anesthetics such as Lidocaine and Bupivacaine, Ropivacaine seems to be characterized by a minor cytotoxic effect on muscle cell viability rate [11]. Specifically, an increase in the concentration of Bupivacaine (> 0.1%) led to a significant decrease in survival of primary cultures of myoblasts isolated from mice and in cultures of immortalized myoblasts, while a concentration of Lidocaine ≥ 0.08% resulted in a drastic decrease in cell viability of the C2C12 myogenic precursor cell line. In contrast, our results, conducted on primary cultures of human myoblasts, showed that the cytotoxic effect of this drug is not only dose-dependent, but also causes increased levels of oxidative stress, and impairment of the myogenic differentiation pathway, leading to altered expression of the myogenic markers MyoD and Myf5.

Noteworthy, our results provide further support to what has already been stated in the literature, about the tissue damage that is caused by the administration of a local anesthetic on muscle. The triggering of the inflammatory process with consequent increase in oxidative stress observed, if not counteracted, may result in the alteration of some molecular pathways leading to the release of harmful molecules even for bone tissue, given its close proximity to muscle tissue. Therefore, the use of local anesthetics, by compromising the quality of muscle tissue, could indirectly compromise the quality of bone tissue as well and hinder the proper healing process of a fragility fracture, both because good muscle mass is necessary to allow functional recovery of the fractured patient after surgery and because the alteration of tissue homeostasis of muscle, caused by oxidative stress, could alter the flow of myokines and osteokines between the two tissues.

## Conclusions

OP and the resulting fragility bone fractures are a significant public health problem, placing significant socioeconomic burdens on those affected and the community. For patients undergoing total or partial hip arthroplasty surgery for the treatment of fragility fractures, an important rehabilitation pathway is needed after surgery Our preclinical findings in vitro results suggest that preventive and parallel treatment with AsA, a common natural nutraceutical, may represent a valid strategy to mitigate the negative effects exerted by Ropivacaine in the clinical practice, and in general to improve full functional muscle recovery of patients undergoing hip replacement surgery for OP fracture.

## Electronic supplementary material

Below is the link to the electronic supplementary material.

Supplementary Material 1

## Data Availability

The data that support the findings of this study are available from the corresponding author, who is Dr Maria Giovanna Scioli, PhD, Anatomic Pathology Division, Department of Biomedicine and Prevention, University of Rome Tor Vergata, upon reasonable request.
