# Supplementary figures and images for: Ascorbic acid reduces Ropivacaine-induced myotoxicity in cultured human osteoporotic skeletal muscle cells
Source: BMC Musculoskelet Disord. 2023 Jul 15;24:576. doi: 10.1186/s12891-023-06702-5 (PMC10350256; doi:10.1186/s12891-023-06702-5)

## Nox4

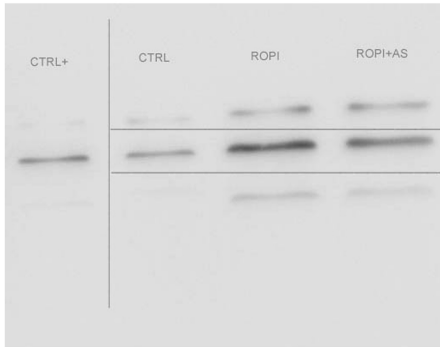

## GAPDH

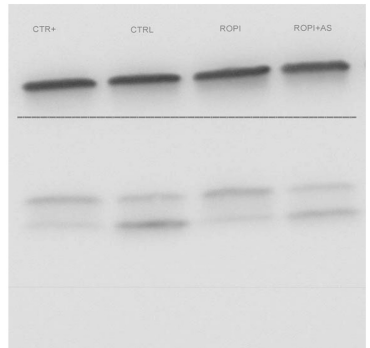

## Myostatin-GAPDH

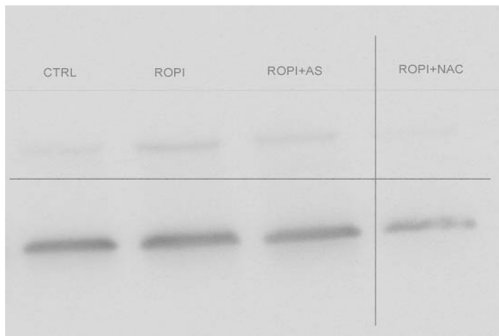

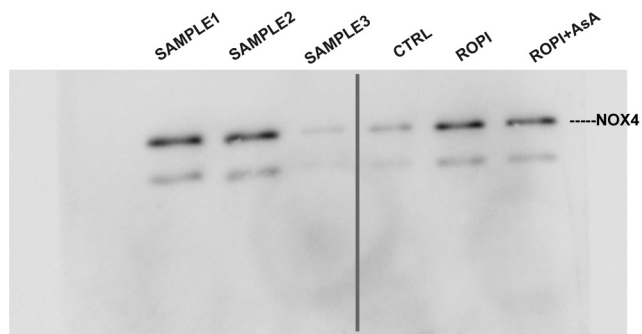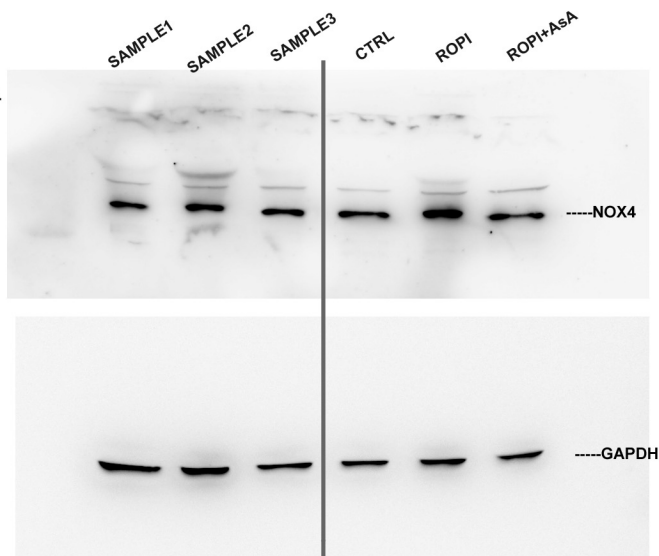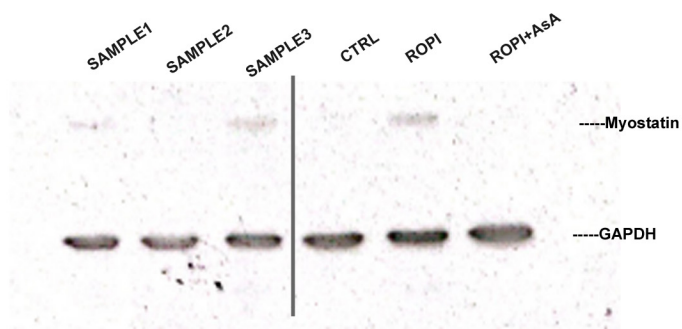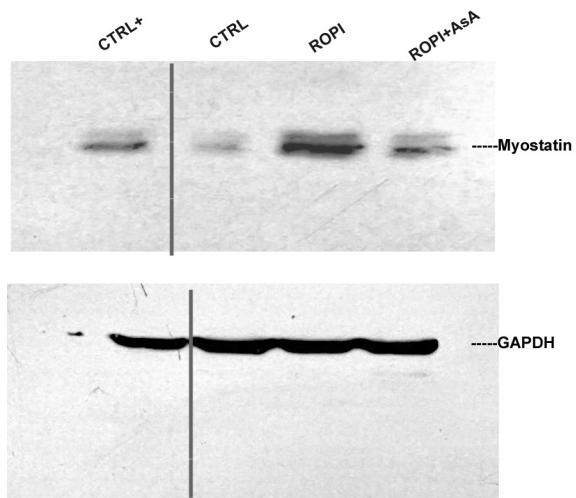

**Blot replicates**

Supplement: Supplementary file 1 — Supplementary Material 1 [file 12891_2023_6702_MOESM1_ESM.pdf]
